# The Central Committee's Bill and Nurses in Practice

**Published:** 1919-06-21

**Authors:** 


### THE REGISTRATION OF NURSES.

### The Central Committee's Bill and Nurses in Practice.

THE writer of the article in the Supplement to last week's *British Journal of Nursing* makes a forlorn effort to confuse the issue raised by the changes made in the sub-section of the Central Committee's Bill dealing with the registration of nurses in practice at the time the Act comes into force. What has made nurses suspicious that V.A.D.s and other even less-perfectly-trained women may obtain access to the Register is that whilst the original clause specifies training plus three years in *bona-fide* practice, the amended clause leaves out any reference to training, and substitutes for it the requirement that a nurse shall produce evidence satisfactory to the Council of "having been for at least three years in *bona-fide* practice as a nurse in attendance upon the sick, and as to the conditions under which she was so engaged." That this change is of significant importance is in effect admitted by the writer of the article when she states that the secretaries of the Central Committee are taking steps to have the word "training" reintroduced into their Bill, whilst at the same time characteristically placing upon the Local Government Board the responsibility of the omission.

The College Bill, which, as its promoters contend, confines itself to essentials and avoids unnecessary detail, simply states that a nurse must be qualified for registration "under such conditions as may be prescribed by the rules under this Act." Nurses may rest in confidence that the rules are likely to be sufficiently protective when they note that in the College Bill two-thirds of the Provisional Nursing Council is appointed in equal numbers by the Council of the College and by the Central Committee itself.

